# Phytochemical Composition and Biological Activity

**DOI:** 10.3390/plants13030331

**Published:** 2024-01-23

**Authors:** Antonella Fais, Benedetta Era

**Affiliations:** Department of Life and Environmental Sciences, University of Cagliari, SS 554-Bivio per Sestu, 09042 Monserrato, Cagliari, Italy

Phytochemicals are bioactive plant compounds that provide humans with health benefits, representing a valuable source of novel bioactive molecules.

These compounds are responsible for the plants’ unique color, flavor, and scent. Phytochemicals are essential for the plant’s survival and protect against various diseases and pests. Environmental factors, like the soil type, altitude, and light, impact the phytochemical composition of plants [1]

Medicinal plants’ exposure to abiotic stresses alters their physiology, morphology, and the biosynthesis of secondary metabolites.

UV-B radiation, which is a major abiotic stress factor, can increase the concentration of bioactive components in medicinal plants. This radiation can be utilized as a straightforward and eco-friendly technique to enhance the concentration of bioactive components in medicinal plants. An excellent abiotic stress-inducing strategy to accelerate the accumulation of secondary metabolites is the use of methyl jasmonate. This was evident when *Tagetes patula* L. petals were treated with ultraviolet-B irradiation and methyl jasmonate, which resulted in changes in the quercetin derivatives and antioxidant activity [2].

Several studies have revealed that these phytochemicals possess numerous therapeutic benefits and can aid in preventing various diseases, including cancer, cardiovascular, inflammatory diseases, and neurodegenerative disorders.

For instance, plant flavonoids have bioactive properties that provide humans with many health benefits. The extracted flavonoids are crucial in developing new drugs used to treat inflammatory disorders. A study was conducted on *Passovia ovata*, a neotropical mistletoe species, to investigate the in vitro and in vivo phytochemical and immunomodulatory properties using cultured RAW 264.7 cells and murine models. According to its phytochemical profile, this plant has many flavonoids and secondary compounds with great pharmacological potential. *P. ovata* demonstrates a high concentration of flavonoids and prominent effects in reducing pro-inflammatory markers in vitro and inhibiting paw oedema [3].

These compounds also improve immune functions, promote healthy ageing, and enhance people’s well-being.

The polyphenol compounds detected in the chemical profile of *Crataegus laciniata* could be linked to the biological properties of the plant’s flowers [4]. *C. laciniata* flowers (CLF) are a rich source of bioactive compounds, which could be used to treat metabolic disorders and skin hyperpigmentation.

Furthermore, the phenolic and flavonoid contents confirm that ethanolic extract is rich in flavonoids.

The inhibition of α-amylase and α-glucosidase by CLF ethanol extract could be related to its phytochemical composition; it is rich in glycosides, such as hyperoside and vitexin.

Considering that hyperoside and vitexin are tyrosinase inhibitors, their involvement also in the antimelanogenic activity of CLF should be regarded.

Tests on antimelanogenic activity were performed in vitro on the tyrosinase enzyme and in vivo on zebrafish embryos.

In some instances, phytochemical characterization can be useful in the classification of taxa. The chemotaxonomy and the classical morpho-anatomical method yield a much clearer insight into the phylogenetic relationships of the taxa that proved to be problematic from the morpho-anatomical standpoint, such as *Dianthus*. Mladenovic et al. identified 389 wax constituents, including 18 new natural products. The multivariate statistical analysis (MVA) of wax composition data for seven different *Dianthus* taxa, including a wax sample for a taxon belonging to another closely related genus of the Caryophyllaceae family (*Petrorhagia prolifera*), produced information about the composition of plant waxes of dianthus and the related genera [5].

Numerous studies have highlighted the pharmacological effects of plant extracts, such as a study conducted on *Polystichum lonchitis* L., a fern belonging to the family Dryopteridaceae. Among the pharmacological activities, the most relevant effects were observed on the analgesic and anti-inflammatory activities, followed by the antipyretic and antispasmodic activities. Furthermore, methanolic and aqueous extracts have antioxidant properties. Some bioactive compounds, such as α-D-Galactopyranoside and methyl and *n*-hexadecanoic, have been isolated, providing the basis to evaluate new drugs with a curative potential and fewer side effects [6].

Plant extracts have various beneficial properties, including antimicrobial effects, as shown in the response of chestnut extract and its components to *Staphylococcus aureus*. The MIC values of various isolated compounds were identified by determining the bioactive compounds and their contribution to antimicrobial activity, with gallic acid exhibiting the highest MIC values [7].

In the plant kingdom, some species are underutilized and could be a source of nutritionally relevant compounds. For example, Arecaceae seeds are fruit by-products whose valorization is desirable. Rincón-Cervera and co-workers [8] have studied the seeds of 24 Arecaceae taxa for fatty acids and phenolics and their antitumor activity against the colorectal cancer cell line. The study results indicated that seeds of the Arecaceae plant family could be a cost-effective source of beneficial compounds that support people’s health.

In a previous research study, the impact of extracts was analyzed using animal models. A rat model of prostate cancer was used to study the effects of an extract of *Pueraria lobata* roots rich in isoflavones and *Phaffia rhodozyma* extract rich in astaxanthin [9].

Throughout history, people have used plants in traditional medicine to treat various ailments. Considering this, the role of secondary metabolites in various species is relevant. The purpose of specific reviews is to provide an overview of the role of specific metabolites. For example, a review of the medicinal properties of the *Myristica* genus was conducted. Another review focuses on the distribution of 25 acylphenols and dimeric acylphenols in the *Myristica* genus. This covers their extraction, isolation, and characterization among the respective Myristica species, discussing the structural similarities and differences within and between each group of acylphenol and dimeric acylphenol. Finally, the review also examines these compounds’ in vitro pharmacological activities [10].

One of the most crucial research areas in the modern food industry is the extraction of bioactive compounds from plants. Several significant improvements have been made in the bioactive compound extraction field.

The conventional extraction methods often involve using a large amount of organic solvents, which can be harmful to the environment and expensive to buy and dispose of.

Developing and improving more efficient and eco-friendly extraction techniques has been the focus in recent years to overcome the deficiencies of the conventional method.

These eco-friendly extraction techniques can result in selective methods of extracting targeted bioactive compounds, as reported for edible feijoa flowers [11]. Among the eco-sustainable extractions, steam distillation has allowed to obtain essential oils from *Hedyosmum cumbalense* and *Hedyosmum spectabile* with an anticholinesterase potential [12].

Essential oils are used in various consumer products, such as detergents, soaps, cosmetics, pharmaceuticals, perfumes, food, drinks, and insecticides. Due to resistance development, chemical insecticides are losing their effectiveness, and plant metabolites are gaining recognition as potential alternatives. Zhang et al. [13] demonstrated that *Perilla frutescens* oil, including its main component, 2-hexanoylfuran, has insecticidal effects on *Culex pipiens pallens* mosquitoes, indicating its potential as a new plant-based biopesticide.

This Special Issue explores different plant extracts’ phytochemical composition and biological activity. It covers several important aspects, such as changing the concentration of secondary metabolites, various extraction methods, and the pharmacological properties of different plant extracts. The information presented in this Special Issue will provide the scientific community with valuable knowledge, helping them better understand and utilize plants for therapeutic purposes.

## References

[1] Abbas F., Zhou Y., O’Neill Rothenberg D., Alam I., Ke Y., Wang H.C. (2023). Aroma Components in Horticultural Crops: Chemical Diversity and Usage of Metabolic Engineering for Industrial Applications. Plants.

[2] Kim J.H., Duan S., Lim Y.J., Eom S.H. (2022). Changes in Quercetin Derivatives and Antioxidant Activity in Marigold Petals (*Tagetes patula* L.) Induced by Ultraviolet-B Irradiation and Methyl Jasmonate. Plants.

[3] Magalhães I.d.F.B., Figueirêdo A.L.M., da Silva E.M., de Miranda A.A.B., da Rocha C.Q., da Silva Calabrese K., Almeida-Souza F., Abreu-Silva A.L. (2023). Effects of *Passovia ovata* Mistletoe on Pro-Inflammatory Markers In Vitro and In Vivo. Plants.

[4] Mirabile S., D’Angelo V., Germanò M.P., Arabi S.P., Parisi V., Raimondo F.M., Rosa E. (2024). Chemical Profile and Health-Promoting Activities of *Crataegus laciniata* (Rosaceae) Flowers. Plants.

[5] Mladenović M.Z., Ristić M.N., Bogdanović A.I., Ristić N.R., Boylan F., Radulović N.S. (2023). Wax Composition of Serbian *Dianthus* Spp. (Caryophyllaceae): Identification of New Metabolites and Chemotaxonomic Implications. Plants.

[6] Sher J., Jan G., Israr M., Irfan M., Yousuf N., Ullah F., Rauf A., Alshammari A., Alharbi M. (2023). Biological Characterization of *Polystichum lonchitis* L. for Phytochemical and Pharmacological Activities in Swiss Albino Mice Model. Plants.

[7] Štumpf S., Hostnik G., Langerholc T., Pintarič M., Kolenc Z., Bren U. (2023). The Influence of Chestnut Extract and Its Components on Antibacterial Activity against *Staphylococcus aureus*. Plants.

[8] Rincón-Cervera M.Á., Lahlou A., Chileh-Chelh T., Lyashenko S., López-Ruiz R., Guil-Guerrero J.L. (2023). Arecaceae Seeds Constitute a Healthy Source of Fatty Acids and Phenolic Compounds. Plants.

[9] Semenov A.L., Tyndyk M.L., Von J.D., Ermakova E.D., Dorofeeva A.A., Tumanyan I.A., Radetskaya E.A., Yurova M.N., Zherebker A., Gorbunov A.Y. (2023). Effects of Isoflavone-Rich NADES Extract of *Pueraria lobata* Roots and Astaxanthin-Rich *Phaffia Rhodozyma* Extract on Prostate Carcinogenesis in Rats. Plants.

[10] Othman M.A., Sivasothy Y. (2023). Acylphenols and Dimeric Acylphenols from the Genus *Myristica*: A Review of Their Phytochemistry and Pharmacology. Plants.

[11] Gil K.A., Jokić S., Cikoš A.M., Banožić M., Jakovljević Kovač M., Fais A., Tuberoso C.I.G. (2023). Comparison of Different Green Extraction Techniques Used for the Extraction of Targeted Flavonoids from Edible Feijoa (*Acca sellowiana* (O.Berg) Burret) Flowers. Plants.

[12] Guerrero A., Guerrero E., Cartuche L., Cumbicus N., Morocho V. (2023). Chemical Profiling of *Hedyosmum cumbalense* and *Hedyosmum spectabile* (Chloranthaceae) Essential Oils, and Their Antimicrobial, Antioxidant, and Anticholinesterase Properties. Plants.

[13] Zhang R., Zhang W., Zheng J., Xu J., Wang H., Du J., Zhou D., Sun Y., Shen B. (2023). Toxic Effects of *Perilla frutescens* (L.) Britt. Essential Oil and Its Main Component on Culex Pipiens Pallens (Diptera: Culicidae). Plants.

